# ClonoCluster: A method for using clonal origin to inform transcriptome clustering

**DOI:** 10.1016/j.xgen.2022.100247

**Published:** 2023-01-12

**Authors:** Lee P. Richman, Yogesh Goyal, Connie L. Jiang, Arjun Raj

**Affiliations:** 1 Department of Pathology, Brigham and Women’s Hospital, Boston, MA, USA; 2 Department of Genetics, Perelman School of Medicine, University of Pennsylvania, Philadelphia, PA, USA; 3 Department of Bioengineering, School of Engineering and Applied Sciences, University of Pennsylvania, Philadelphia, PA, USA; 4 Department of Cell and Developmental Biology, Feinberg School of Medicine, Northwestern University, Chicago, IL, USA; 5 Center for Synthetic Biology, Northwestern University, Chicago, IL, USA; 6 Genetics and Epigenetics, Cell and Molecular Biology Graduate Group, Perelman School of Medicine, University of Pennsylvania, Philadelphia, PA, USA

**Keywords:** single cell, Bioinformatics, lineage tracing, clonality, clustering

## Abstract

Clustering cells based on their high-dimensional profiles is an important data reduction process by which researchers infer distinct cellular states. The advent of cellular barcoding, however, provides an alternative means by which to group cells: by their clonal origin. We developed ClonoCluster, a computational method that combines both clone and transcriptome information to create hybrid clusters that weight both kinds of data with a tunable parameter. We generated hybrid clusters across six independent datasets and found that ClonoCluster generated qualitatively different clusters in all cases. The markers of these hybrid clusters were different but had equivalent fidelity to transcriptome-only clusters. The genes most strongly associated with the rearrangements in hybrid clusters were ribosomal function and extracellular matrix genes. We also developed the complementary tool Warp Factor that incorporates clone information in popular 2D visualization techniques like UMAP. Integrating ClonoCluster and Warp Factor revealed biologically relevant markers of cell identity.

## Highlights

• Clonal information represents an alternative clustering approach to transcriptomes
• *ClonoCluster* combines transcriptomic and clonal data to generate hybrid clusters
• Incorporating clonal data rearranges clusters via ECM and ribosomal gene expression
• Hybrid clusters identify biologically relevant markers and reduce cell type entropy

## Graphical abstract

**Figure:**
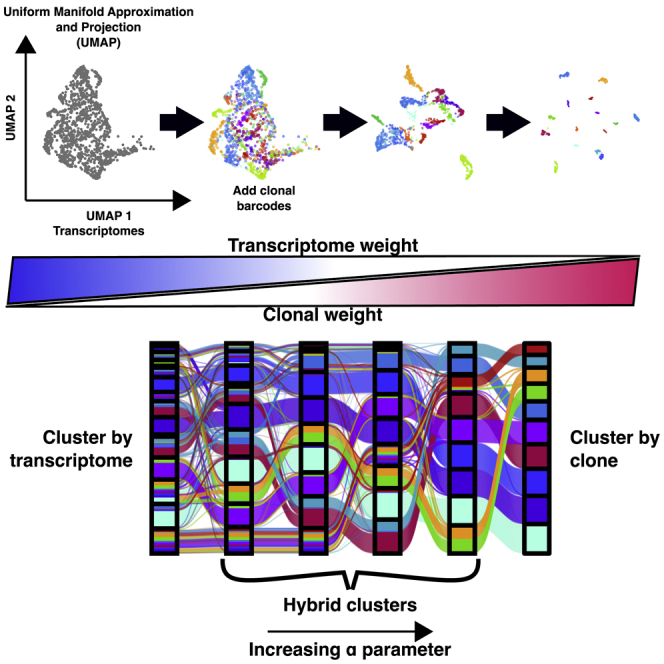

## Teaser

Novel cellular barcoding methods in single-cell RNA sequencing provide simultaneous clustering substrates: transcriptomic and clonal information. Richman et al. developed *ClonoCluster* to combine these approaches to produce hybrid clusters. Hybrid clusters are separated by biologically significant markers and reduce entropy of labeled cell types across clusters.

## Introduction

Since the advent of high-dimensional molecular profiling, clustering has been the most common form of data analysis and visualization applied, allowing one to form groups out of entities with similar profiles.^1^^,^^2^ Clustering has enabled the detection of networks of disease genes and loss of function patterns across cancer cell lines.^3^^,^^4^ More recently, the development of single-cell measurement technologies has allowed the high-dimensional profiling of individual cells. In this context, clustering has been used to categorize cells into discrete molecular states, often termed “fates” or “types,” that can be associated with distinct cellular functions.^5^^,^^6^ At the same time, in many biological contexts, single cells also have lineage relationships; i.e., they may arise from a common progenitor. This information in principle provides a complementary way to cluster profiles of single cells, but it has not been incorporated into the systematics of single-cell profiles.^7^

Clustering purely based on molecular profiling has been successful in many instances but relies on a number of assumptions that have been difficult to evaluate rigorously. Briefly, most clustering approaches start with some form of feature selection or dimensionality reduction. Data points are then grouped to minimize the distances between elements of the same groups, by k-means clustering, hierarchical clustering, or graph-based community detection methods that use connectivity to inform group membership.^5^^,^^6^^,^^8^ Intrinsic to all these methods is the use of some sort of distance between cells measured by some metric of distance between their molecular profiles, but in principle many other types of information could be used to inform or modify these distances.

Recently, the development of cellular barcoding systems has provided an alternative means by which to group cells. Primarily, these systems have been used for the longitudinal tracking of molecular profiles through some sort of biological process, such as differentiation or therapy resistance.^9^^,^^10^^,^^11^^,^^12^^,^^13^^,^^14^^,^^15^ Strikingly, these studies have concluded, in some instances, that single-cell differences in the expression of few or even just one gene can predict the ultimate fate of the cell, differences that would not have been detected by standard clustering algorithms. For example, this approach identified the expression of *Mettl7a1*, a methyltransferase, as a driver of successful stem cell reprogramming,^13^ identified TCF15 as necessary and sufficient for hematopoietic stem cell self-renewal, and identified *Pou2f2* expression in progenitor cells as predicting DC-like versus neutrophil-like monocyte fates.^12^^,^^16^^,^^17^ Such results suggest that the incorporation of clone information could be very useful in determining the expression differences between cells that have distinct functional outcomes. Being able to weigh both transcriptome and clone information in clustering algorithms would potentially enable the identification of such “hidden” factors.

We thus developed an algorithm that we call “ClonoCluster” that integrates transcriptome and clone barcode information, allowing one to cluster cells using a continuous parameter (α) that adjusts the relative weight of transcriptome versus clone information. We applied ClonoCluster to six previously published independent single-cell RNA sequencing datasets, including *in vitro* hematopoiesis, directed stem cell differentiation, and drug treatment of tumor cell lines.^11^^,^^12^^,^^18^ We found massive rearrangements of the assignments of cells to clusters as α was shifted to weigh clonal origin more heavily. These rearrangements had novel, potentially more biologically interpretable, cluster markers, and were associated with expression of genes involved in extracellular matrix production and translation. These results held across datasets where clone fate was determined intrinsically, and the effects were considerably less strong in the dataset where cell fate was determined extrinsically. Inspired by this clone-weighted network graph clustering approach, we developed a tunable parameter (the Warp Factor, ranging from 0 to 10), that incorporates clonality information into the dimensionality reduction step prior to the commonly used Uniform Manifold Approximation and Projection (UMAP) algorithm for visualizing high-dimensional datasets. We included ClonoCluster and Warp Factor into an open-source R package, *ClonoCluster* (https://github.com/leeprichman/ClonoCluster). As barcoding data become more prevalent, ClonoCluster can provide a means to evaluate the degree to which clustering can be altered by factoring in clonal origin.

### Design

#### ClonoCluster integrates clone barcode and transcriptome information

Clone barcode assignment and transcriptome-level data represent two different modalities of data that can be used to cluster single-cell RNA sequencing. In a prototypic clonal barcoding experiment, a population of cells is transfected with random transcribed barcodes such that each initial clone is likely to express a unique barcode. After proliferation, experimenters apply some additional experimental conditions, such as drug treatment or differentiation.^11^^,^^12^^,^^13^^,^^14^^,^^18^ At the chosen endpoint, one can perform single-cell RNA sequencing on a pool of individual cells, which is itself composed of some number of clones marked by barcodes. The barcodes themselves can be determined by various side reactions and subsequent sequencing, thus adding a clone identifier to each cell’s transcriptome. (In practice, technical constraints on clone identification and sampling for single-cell RNA sequencing mean that only some subsets of sequenced cells will have an identifiable barcode.)

**Figure 1:**
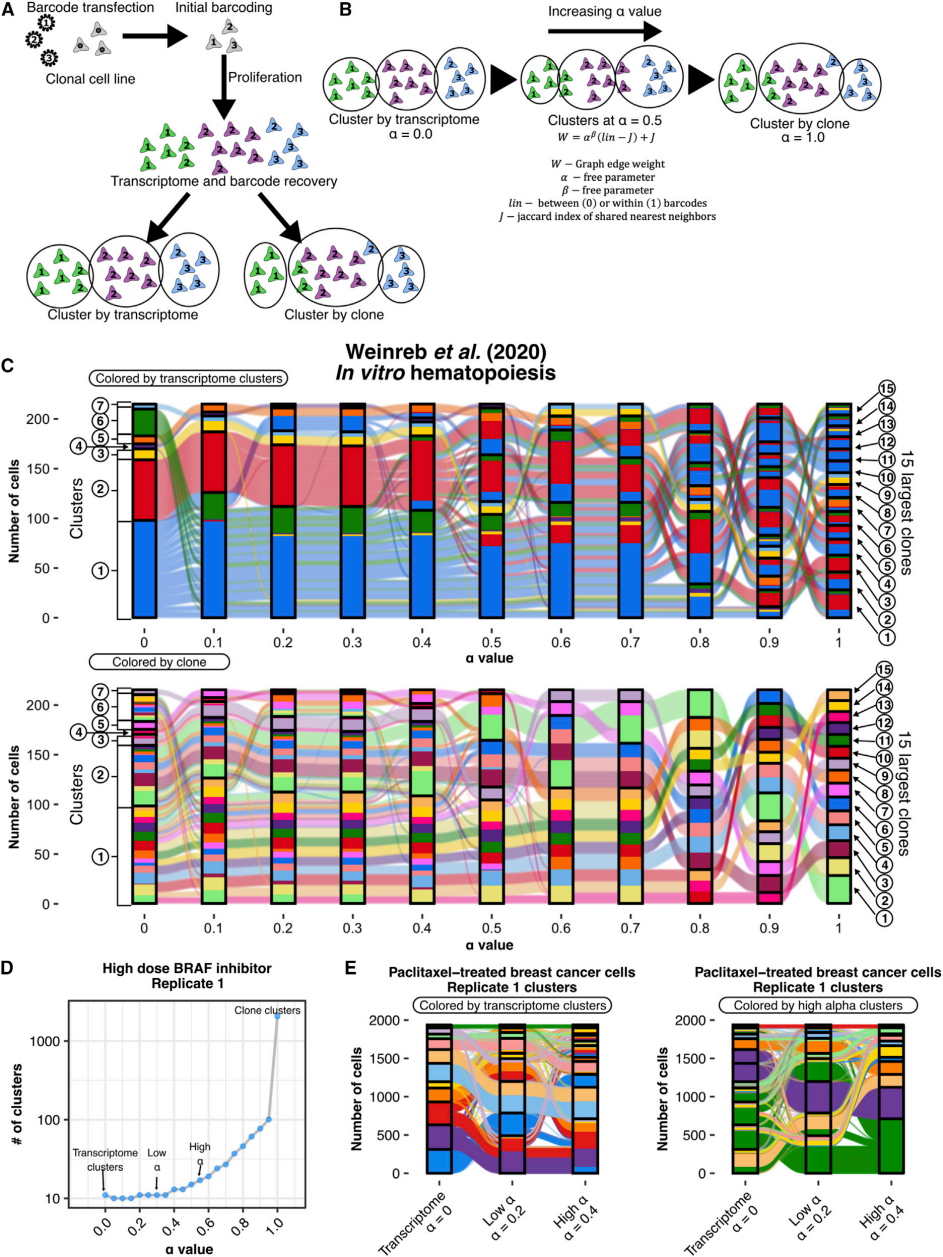
The ClonoCluster method integrates transcriptome and clone clustering modalities in a tunable manner using the α parameter (A) Schematic depicting a generic approach to single-cell barcoding that yields output clusterable by two modalities of data, transcriptome clustering, and clone clustering by recovered barcode. (B) Schematic depicting the integration of these clone and transcriptome clustering modalities using the ClonoCluster method, in which the transcriptome nearest neighbor network graph edge weights are modified to incorporate clone clusters with a tunable free parameter, α. At α = 0, clustering is identical to traditional transcriptome clusters. At α = 1, clusters are consistent with clone barcode assignments. (C) Sankey diagram depicting the reorganization of cell clusters from the 15 largest clone clusters present at day 2 in an *in vitro* hematopoiesis assay^12^ with increasing α value colored by initial transcriptome cluster (top) and clone (bottom). Nodes/boxes represent clusters and ribbons depict the flow of cells between clusters. (D) Representative plot of high-dose BRAF inhibitor-treated melanoma clonal cell line (clonal WM989 cells treated with 1 μM of the BRAF inhibitor vemurafenib^11^) showing that cluster number approaches unique clone barcode number with increasing α value at fixed community detection resolution. “High α” values were chosen based on the approximate shoulder of the curve after which clusters rapidly break into individual clone clusters with increasing α. “Low α” values were determined by the half “high α” value rounded to the nearest 10th value. At α = 0, clustering is identical to traditional transcriptome clusters. At α = 1, clusters are consistent with clone barcode assignments. (E) Representative Sankey diagram of the 15 largest clone clusters from the high-dose BRAF inhibitor dataset treated melanoma clonal cell line depicting rearrangements of clusters at transcriptome, low α, and high α levels, colored by initial transcriptome cluster assignment (left) and high α cluster assignment (right).

Once cells have both transcriptome and clone information attached to them, one can compare methods of classification. Two popular software packages for classifying cells by transcriptome information alone are *Seurat* and *scanpy*,^19^^,^^20^ both of which apply community detection algorithms to network graphs to identify the most interconnected cell clusters. We can then directly compare the classification of cells by transcriptome clusters versus clone barcodes (Figure 1A). In principle, these two classification schemes could be virtually identical, or they could be completely uncorrelated with each other.

#### The tunable parameter α yields hybrid clone-transcriptome defined clusters

We wondered whether there was some way to incorporate both clone and transcriptome information to generate “hybrid” clusters that group cells that balance transcriptomic similarity and clonal relationships. In order to generate such hybrid clusters, we developed the ClonoCluster model for measuring similarity between cells. This model includes a tunable parameter, α, that shifts weight between clustering by cell transcriptomes alone (α = 0) and “clustering” by clone barcode alone (α = 1) (Figure 1B). In well-established single-cell RNA sequencing analysis packages like *scanpy* and *Seurat*, the algorithms build a network graph of cells (nodes) connected by edges that are weighted by transcriptional similarity (“transcriptome weight”), determined by the number of shared nearest neighbors in principle component space.^19^^,^^20^ The clustering itself is then determined by community detection within this graph, returning the most highly interconnected groupings of cells as the assigned clusters. In ClonoCluster, we retained this overall structure, incorporating clone information by modifying the weights as follows. For each edge between cells, we also created a “clone weight” of 1 or 0 based on whether the cells have the same or different barcodes. We then normalized the “clone weight” by the number of cells with that barcode to ensure that the attractive “force” of barcodes did not scale with the number of cells. We linearly combined transcriptome and clone weights using α such that it returned purely transcriptome weights for α = 0 and purely clone weights for α = 1 (Figure S1A). We could then use this graph for cluster assignment just as is done by the conventional algorithms (Figure S1B). Thus, for values of α in between 1 and 0, ClonoCluster provides hybrid clusters that weigh both clone and transcriptome information.

## Results

### Clone-to-cluster concordance variably increases with α

We used ClonoCluster to generate hybrid clusters from six different clone barcoded single-cell RNA sequencing datasets from our lab and others^11^^,^^12^^,^^18^ (see STAR methods and Table 1). We first performed hybrid clustering at stepwise values of α from traditional transcriptome-only clusters (α = 0) to the clone groupings (α = 1). We wanted to determine how distinct hybrid clusters were from transcriptome or clone clustering alone as well as how increasing α reorganized clusters from initial transcriptome clusters to individual clone clusters. In order to visualize the flow of cells through these progressive hybrid clusters, we constructed Sankey diagrams^21^ of the datasets using the 15 largest clone clusters per sample (Figures 1C and S2A). There was visible reorganization of cells throughout clusters among the top 15 clone clusters as α increased, with differences in cluster number, composition, and size throughout much of the range of α in all samples. As expected, when α approached 1, the concordance between hybrid clusters and clone clusters increased, meaning that hybrid clusters were largely composed of individual clones or combinations thereof, with the frequency of clones being split across clusters becoming less. (At α = 1, clusters were equivalent to clone groupings.) Within each dataset, cells with the same clonal origin (i.e., sharing a barcode) demonstrated variable degrees of reorganization into concordant clusters at given levels of α, with some requiring higher α values to completely unify into a single hybrid cluster than others.

**Table 1:** Dataset descriptions and sources

Dataset name	Source	Description	No. of Replicates	Fate determination
Low-dose BRAF inhibitor	Goyal et al. *bioRxiv* (2021)^11^	WM989 A6-G3 clonal melanoma cells treated with 100 nM vemurafenib for 3–4 weeks	1	Intrinsic
High-dose BRAF inhibitor	Goyal et al. *bioRxiv* (2021)^11^	WM989 A6-G3 clonal melanoma cells treated with 1 μM vemurafenib 3–4 weeks	2	Intrinsic
Cario-directed iPSCs	Jiang et al. *Genome Biol.* (2022)^18^	PENN123i-SV20 human IPSC line directed toward cardiomyocyte fate sequenced on day 14	1	Extrinsic
*In vitro* hematopoiesis	Weinreb et al. *Science* (2020)^12^	Hematopoietic progenitor cells derived from murine bone marrow differentiating in culture for 2 days	1	Intrinsic
WM983B BRAF inhibitor	Goyal et al. *bioRxiv* (2021)^11^	WM983B E6-C6 clonal melanoma cells treated with 100 nM vemurafenib for 3–4 weeks	2	Intrinsic
Paclitaxel-treated breast cancer cells	Goyal et al. *bioRxiv* (2021)^11^	MDA-MB-231D4 clonal breast cancer cells treated with 1 nM paclitaxel for 3–4 weeks	2	Intrinsic

The patterns of rearrangement also varied between datasets. For example, the cardio-directed iPSC dataset showed less concordance between clone clusters and transcriptome clusters among the top 15 largest clone clusters compared with WM989 A6-G3 melanoma cells treated with high-dose vemurafenib. In the cardio-directed iPSC dataset, each of the top clone clusters segregated to a separate cluster by α = 0.6, compared with α = 0.8 or 0.9 in the two melanoma replicates (Figure S2A). We formally evaluated clone-to-cluster concordance using Cohen’s κ, a measure of agreement between classifications (grouping by clone versus grouping by assigned cluster) computed from the 2 × 2 confusion matrix of clone barcode and cluster membership. When clonal cells sharing a barcode are more frequently assigned to a single cluster, Cohen’s κ is high. (At α = 1 where hybrid clusters and clonal clusters are equivalent, Cohen’s κ must also equal the maximum value of 1.) For most datasets, we observed a gradual transition of Cohen’s κ from 0 to 1 as α increased, suggesting that there was a graded conglomeration of clone barcodes within hybrid clusters as clonality was increasingly factored into the clustering algorithm. The cardiomyocyte-directed iPSCs, however, displayed a very sudden switch between low κ and high κ (Figure S2B), suggesting that there are few meaningful intermediate hybrid clusters between purely transcriptome clustering and purely clone clustering. Sankey visualization matched this interpretation by showing that clones were quite intermixed between the transcriptome-only clusters, with little obvious areas of partial conglomeration. In the cardiomyocyte-directed iPSC case, biologically, our prior findings suggested that cell fate was extrinsically determined and did not correlate well with clonality; hence, hybrid clusters would increasingly be composed of cells of essentially random types, explaining these observations for this dataset.

As α approaches 1, the number of hybrid clusters identified approaches the large number of individual clone clusters in the dataset, which are generally more numerous than the standard transcriptome clusters returned using common methods. We wondered if we could identify the maximum α value that generates a number of hybrid clusters similar to the number of transcriptome clusters that also showed significant reorganization of cells to be distinct from transcriptome-only clustering. We therefore computed the number of clusters returned in each sample across α values to identify the maximum α value that still returned a number of clusters in this range; the final cluster composition and cluster number was determined by the Louvain algorithm as implemented in the *Seurat* package.^19^^,^^22^ The Louvain algorithm iteratively severs network graph edges to achieve optimal internal interconnectedness of community members (modularity) and returns a greater number of communities at higher values for the input resolution parameter. We performed all clustering at fixed resolution, therefore the rising number of clusters with increasing α value can be attributed to improved modularity of the clusters as networks break apart into isolated networks of like-barcoded clones. “High α” values were chosen based on the maximum value before the inflection point where cluster numbers dramatically increase, with “low α” values being chosen at half this value (Figures 1D and S3). The high α value varied between samples, from 0.4 to 0.7. Even within this restricted range that limited the number of clusters returned, we observed reorganization at low and high α for the top 15 clone clusters (Figure 1E).

### Hybrid α clusters reveal novel cluster markers without loss of marker fidelity

A common goal of single-cell RNA sequencing analysis is the identification of “marker” genes whose expression is high in cells of a particular cluster (i.e., sensitive for cluster membership) and is low in cells in other clusters (i.e., specific for cluster membership).^5^^,^^6^^,^^23^ We consider the most useful markers to be those that are specific and sensitive to the cells in a cluster and thus have “high marker fidelity.” Given that increasing α altered cluster number and membership, we wondered whether the genes that served as the best markers changed with α, and whether those markers were able to maintain similar sensitivity and specificity as they did on purely transcriptomically defined clusters. We used the receiver operating characteristic (ROC) to evaluate all expressed genes as markers to classify cells into clusters at all possible marker expression cutoffs. We used area-under-the-curve (AUC) derived from the ROC to summarize the fidelity of a marker by quantifying its sensitivity and specificity as a marker. An AUC of 0.5 indicates a classifier that is no better than random chance at determining cluster membership of a cell, with a value of 1 being perfectly predictive. We identified the cluster markers with the highest AUC for each hybrid cluster at each α and found that the top markers of the clusters were different for different α values (Figures S4 and S5A).

**Figure 2:**
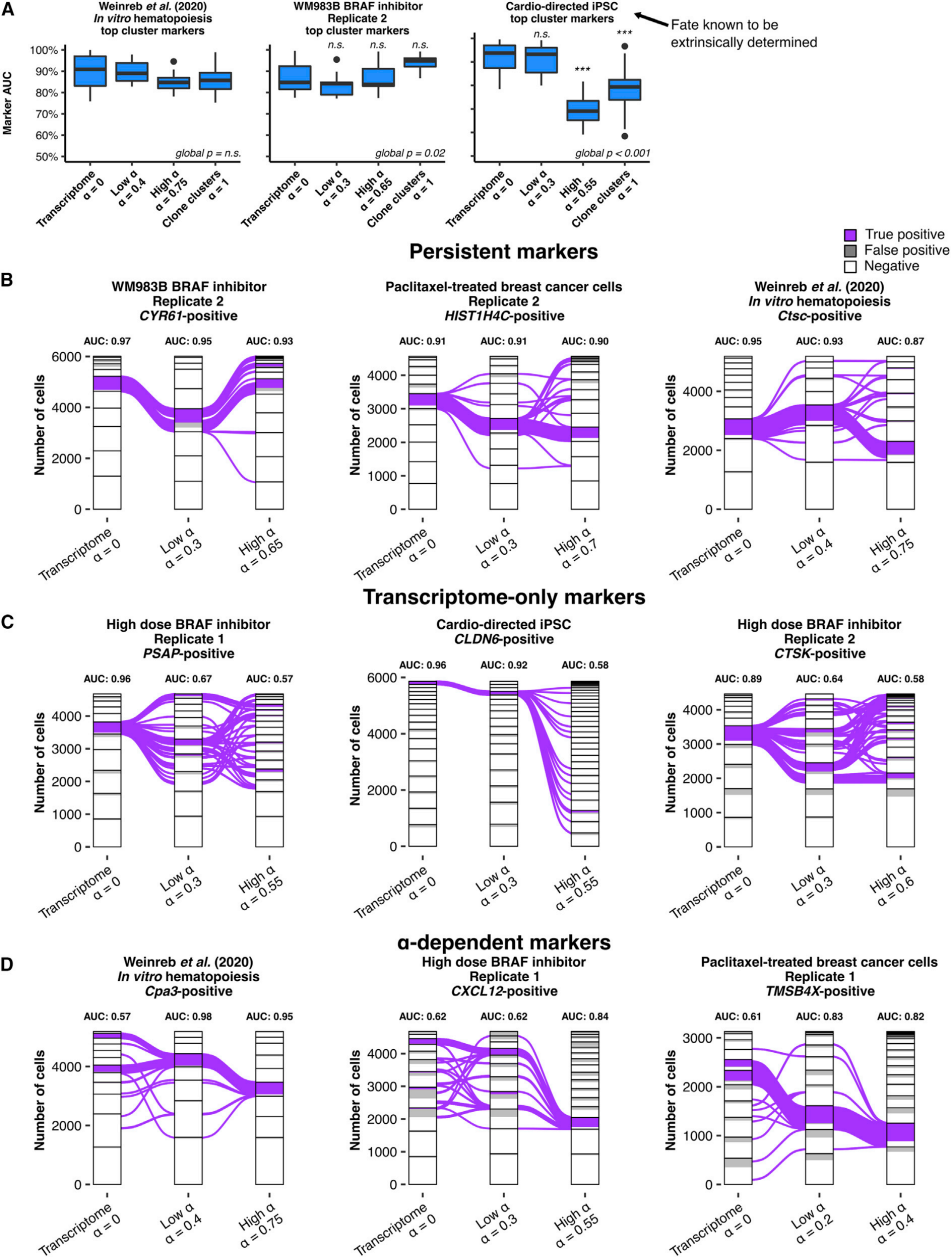
Manipulation of α reveals turnover of cluster markers (A) AUC values for the top marker per cluster at zero, low, high, and maximum (α = 1) values of α for multiple samples, an *in vitro* murine hematopoiesis assay at day 2, a BRAF inhibitor-treated clonal melanoma cell line WM989B, and directed differentiation of induced pluripotent stem cells (iPSCs) toward a cardiomyocyte fate, a system where extrinsic determinants of cell fate are expected be dominant over clone barcodes.^18^ “Global p” indicates the p value for the non-parametric Kruskal-Wallis test with Bonferroni correction. When “global p” was less than 0.05, pairwise Wilcox tests were performed with Bonferroni correction. Annotations above boxes indicate the corrected Wilcox test p value compared with α = 0 (n.s., not significant; ∗∗∗p < 0.001). (B–D) Sankey diagram including all barcoded cells from various samples (see STAR Methods) depicting transcriptome clusters, low α clusters, and high α clusters. Cells positive for the respective marker that are present in the cluster of interest are marked purple (“True Positive”) while cells positive for the marker but not present in the cluster of interest are marked gray (“False Positive”). Positivity thresholds were determined as described in STAR Methods. Area-under-the-curve (AUC) for the marker is annotated above the cluster nodes. Representative markers are chosen to demonstrate markers whose classifier strength persists across α values (B), markers that are only strong in transcriptome clusters (C), and markers that are stronger at high and low α values (D).

With this observed change in top markers, we wondered whether overall fidelity of the top cluster markers at each α, as reflected by AUC, was preserved with hybrid clustering. We found that across almost all datasets, the median AUC of top cluster markers at each value of α were not significantly different from transcriptome clustering alone (Figures 2A and S5B), suggesting that the rearrangements caused by incorporating clone information did not radically decrease the ability to find a single marker for a cluster, despite the fact that the markers themselves changed. We did see decreased marker fidelity in the cardiomyocyte-directed iPSCs at the high α and clonal cluster levels (0.69 and 0.79 versus 0.94). (Low-dose vemurafenib-treated melanoma samples also had a decrease in marker fidelity, although to a lesser extent, 0.80 and 0.84 versus 0.90.) Again, given the extrinsic determination in cardiomyocyte-directed iPSCs, we would expect that markers for hybrid clusters would be hard to find; indeed, many putative markers had poorer performance at high α^18^ (Figure S5A). Thus, in the datasets analyzed in which cell states are thought to be intrinsically determined (Table 1), hybrid clustering yielded new cluster markers revealed by clonality information, often of equal fidelity to transcriptome clustering. The alternative markers identified by ClonoCluster potentially represent genes whose expression varies more between different clonal populations than within a clonal population.

In addition to top cluster markers, we wondered how marker fidelity of any single marker was likely to change as we increased clonal weight with α. We used Sankey plots to directly visualize the flow of marker-positive cells through hybrid clusters as α changed. We found that the fidelity of transcriptome cluster markers varied as α changed, with some markers persisting across all values of α and others changing their fidelity dramatically. Many markers maintained their fidelity over increasing α values (representative samples shown in Figure 2B). We also identified markers that were strong cluster classifiers at the transcriptome cluster level that lost fidelity in low and high α clusters (Figure 2C), suggesting that the classification properties of these markers based on transcriptomes alone was lessened by the addition of clone information. Conversely, we also observed markers that increased in fidelity with increasing α, meaning that those markers were highly expressed in clones that were brought together into a hybrid cluster (Figure 2D). Analysis of marker fidelity across α therefore identified new sets of markers that balance clonal differences with transcriptome differences in cluster assignment, especially favoring markers that are strongly congruent in both.

### Reorganization of clusters with α is explained by differential expression of extracellular matrix and translation-associated genes

**Figure 3:**
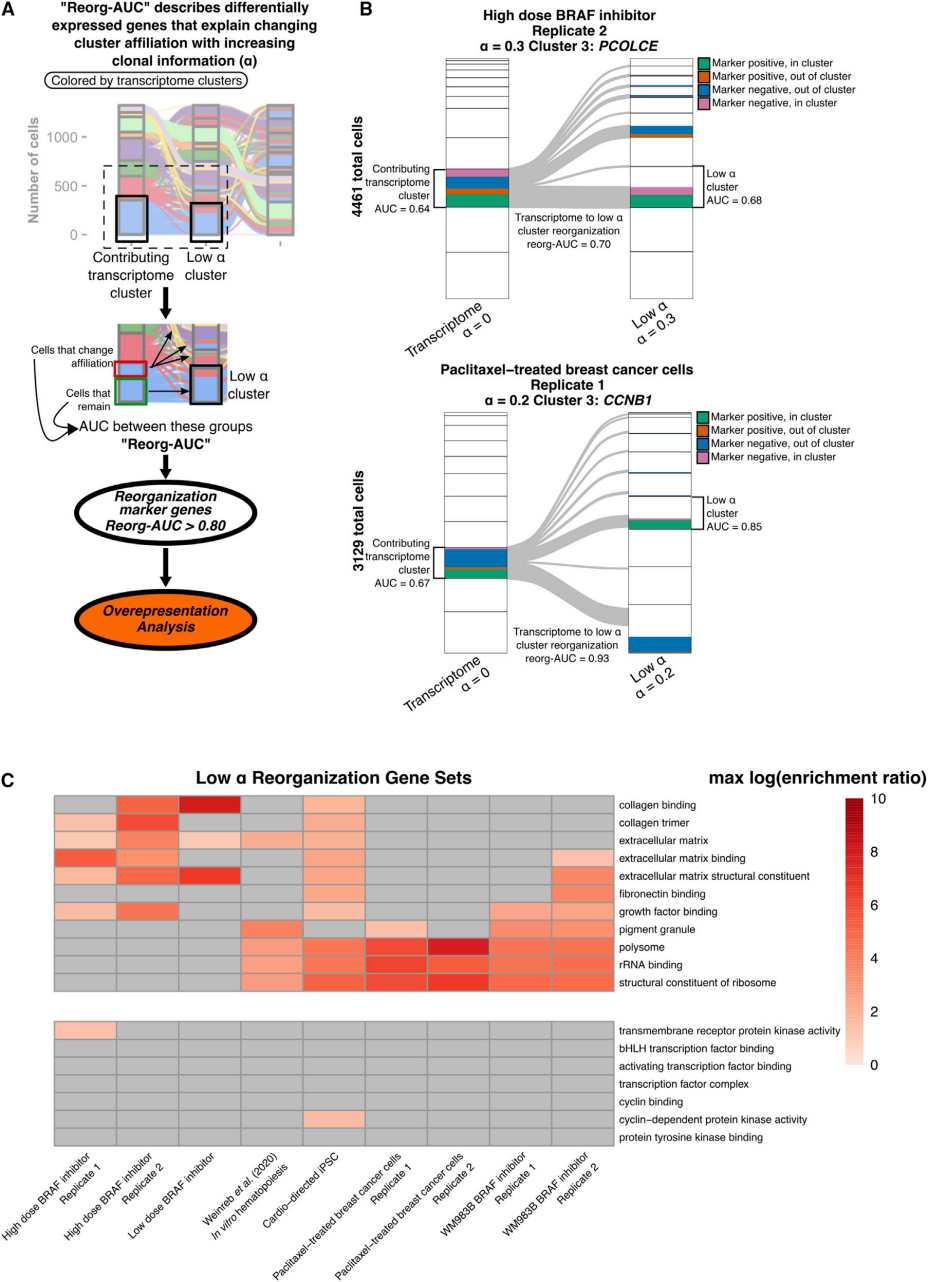
Reorganization markers are enriched in translation and extracellular matrix-associated gene sets (A) Schematic depicting approach to differential expression that explains cluster rearrangements with α modulation. For each hybrid α cluster and contributing transcriptome cluster pair, differential expression analysis is performed between cells inside and outside the α cluster, yielding an AUC for reorganization (reorg-AUC). Geneset overrepresentation analysis was then performed on these groups of differentially expressed genes on reorganizing cells with reorg-AUC >0.80 (see STAR Methods). (B) Representative Sankey diagrams showing a low α cluster and a contributing transcriptome cluster with colors indicating proportion of cells classified by the marker and the associated cluster AUCs and reorg-AUC. (C) Heatmap demonstrating maximum log enrichment ratio for gene sets significantly enriched by overrepresentation analysis of reorganization markers across nine different data sources. All gene sets enriched in three or more samples are shown (top) as well as several commonly explored gene sets as negative controls (bottom). Gray tiles indicate no statistically significant enrichment in the sample (false discovery rate >0.05).

To identify expression of genes associated with reorganization of cells from transcriptome clusters to hybrid clusters, we evaluated genes as classifiers for explaining rearrangement of cells. Figure 3A demonstrates our approach to comparing reorganizing cells to delineate these differentially expressed genes. For all possible transcriptome cluster and low α cluster pairs, we calculated the AUC for each gene in the dataset as a predictor of whether a cell from the transcriptome cluster would become a member of the low α cluster. Genes with high AUC in this analysis were strong predictors of whether or not a cell would switch affiliation to a particular low α cluster, and thus represent the differentially expressed genes that explain cluster rearrangements that either break apart or aggregate cells from the transcriptome clusters upon the incorporation of clonal information to the clustering algorithm. We have termed this AUC of a differentially expressed gene that explains the sorting of clusters into hybrid α clusters as the reorganization AUC (“reorg-AUC”). Any gene with reorg-AUC greater than 0.80 for any transcriptome to hybrid α cluster was considered a “reorganization marker.” Representative examples of markers associated with contributing cells from a transcriptome cluster and a low α cluster of interest are shown in Figure 3B. Reorganization markers were identified in this way for all datasets. Each set of reorganization markers for a paired hybrid cluster and contributing transcriptome clusters was then used to perform an overrepresentation analysis.^24^ Multiple gene sets were significantly enriched in reorganization markers from all samples. For visualization, we generated a heatmap of the maximum enrichment ratio for all gene sets significantly overrepresented in three or more samples, as well as several commonly chosen gene sets to serve as negative controls (Figure 3C). The gene sets shared across the greatest number of samples were associated with translation, “polysome,” “rRNA binding,” and “structural constituent of ribosome,” as well as many samples showing enrichment for genes associated with the extracellular matrix, including “extracellular matrix,” “extracellular matrix structural constituent,” “extracellular matrix binding,” “collagen trimer,” “collagen binding,” and “fibronectin binding.” Enrichment was generally not seen in the negative control gene sets selected. These analyses reveal that the cluster reorganization induced by the incorporation of clonality information, even at low levels of α, are not driven by random gene sets, but rather by specific biological processes, such as those related to the extracellular matrix and translation. Remarkably, these results held across independent datasets taken from very different biological systems, suggesting that the biological processes associated with extracellular matrix and translation may help define a “fingerprint” for clonal information.

### Warp Factor, *s*, modifies UMAP representations to enhance clone separation

**Figure 4:**
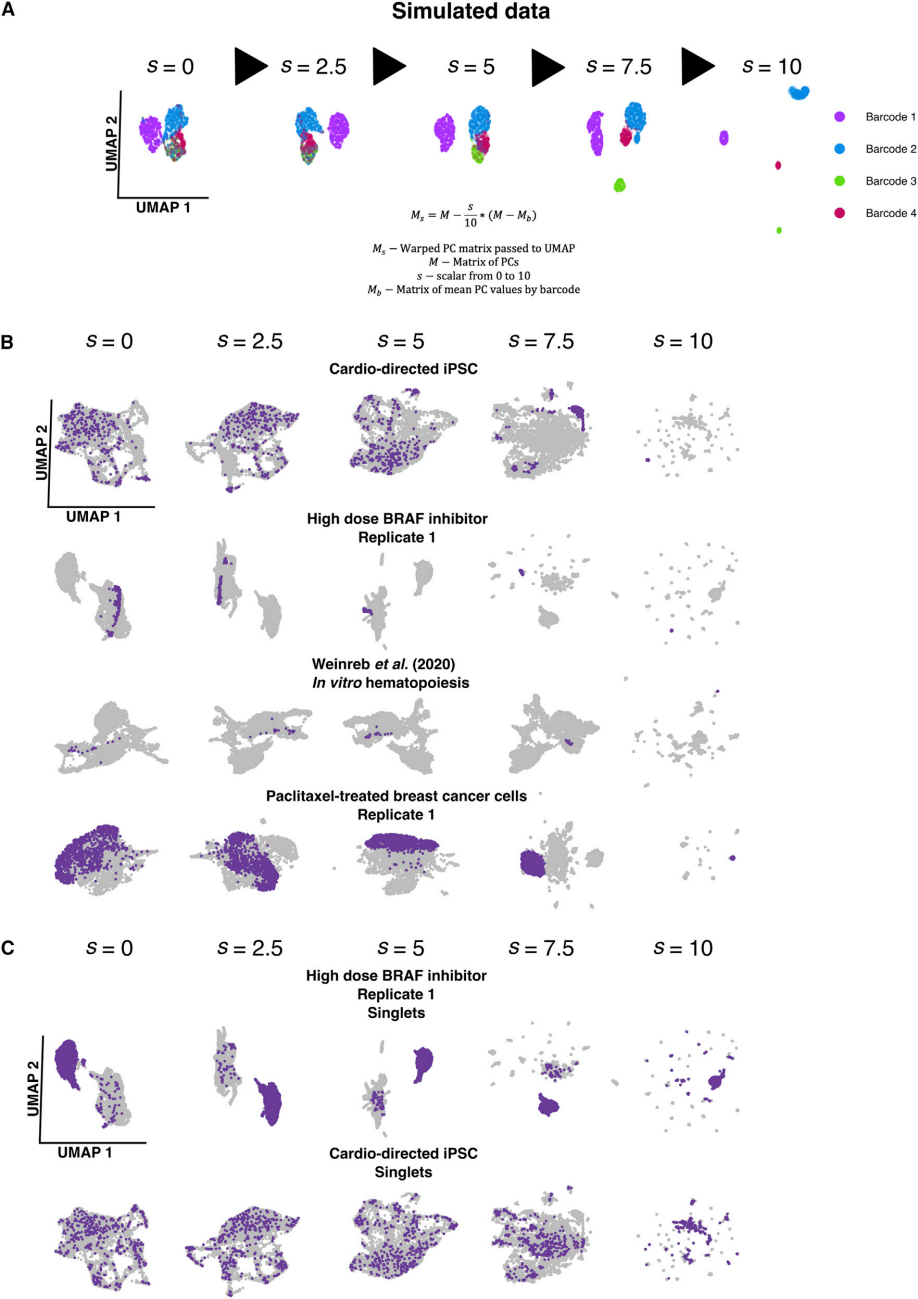
Warp Factor, *s*, is a tunable parameter to modify UMAP visualization to incorporate clone barcode information (A) Demonstration of the effect of increasing Warp Factor (*s*) value on UMAP structure for simulated data of 3,000 cells with four barcodes and six principal components. Each UMAP axis is scaled and centered to allow comparison between facets. (B) UMAPs with increasing Warp Factor for multiple datasets, highlighting a single large clone cluster in each. (C) UMAPs with increasing Warp Factor*s* showing singlets in two datasets. Singlets are cells that are the only cells in the sample with their unique barcode.

In addition to clustering and marker identification, an independent step in single-cell RNA sequencing analysis is two-dimensional visual representation of transcriptome data using dimensionality reduction techniques such as t-SNE or UMAP.^6^^,^^25^ In the same spirit of hybrid clustering, we wondered whether we could apply the principle of adding weighted clone information to these low-dimensional visual embeddings. Whereas α modifies the weight of edges in the network graph input to the clustering algorithm, we sought to construct a method to incorporate clone information with a tunable parameter, the Warp Factor, into the input to the UMAP algorithm. UMAP represents variation in the transcript count matrix-derived principal-component analysis (PCA) in a single manifold using attraction and repulsion components. Two UMAP dimensions are usually projected to visualize the high-dimensional data.^6^^,^^25^ We modified the PCA input to UMAP to incorporate clone barcode information alongside transcriptome variation. To test this approach, we simulated data and used a model to create a modified principal component (PC) matrix in which a tunable “Warp Factor” parameter, *s*, warps the values of PCs for each cell to approach the mean value of the principal component for the clone cluster it belongs to (Figure S6A); i.e., reducing the variance of cells within the clone. At *s* = 0, the PC matrix is unmodified and at the maximum value, *s* = 10, the only variation present in the data will be between clone clusters (Figure S6B). The incorporation of the Warp Factor into visualization promoted separation of clone clusters in UMAP space in simulated data (Figure 4A). We then used the Warp Factor for the visualization of clone clusters in the datasets. As expected, the spread between individual clone clusters reduced across the UMAP with increasing Warp Factor. When we engaged high Warp Factors, individual clone clusters formed distinct spatial clusters in UMAP space. The amount of Warp Factor required to promote distinct separation of individual clone clusters and singlets into isolated spatial groups varied between datasets, likely due to the differences in the size and number of clone clusters as well as initial degree of clone-to-transcriptome concordance (Figure 4B). Unexpectedly, we also observed that “singlets,” cells that are the only member of their clone cluster (have a unique barcode), conglomerated together into a large group, possibly through repulsion from the non-singlet clone clusters (Figure 4C). The Warp Factor method was effective at incorporating clonal information into UMAP visualizations of single-cell RNA sequencing datasets by reducing within-clone cluster variation and increasing between-clone cluster variation in the underlying PC matrix.

### Combining hybrid clustering and Warp Factor highlights unique clusters and markers

**Figure 5:**
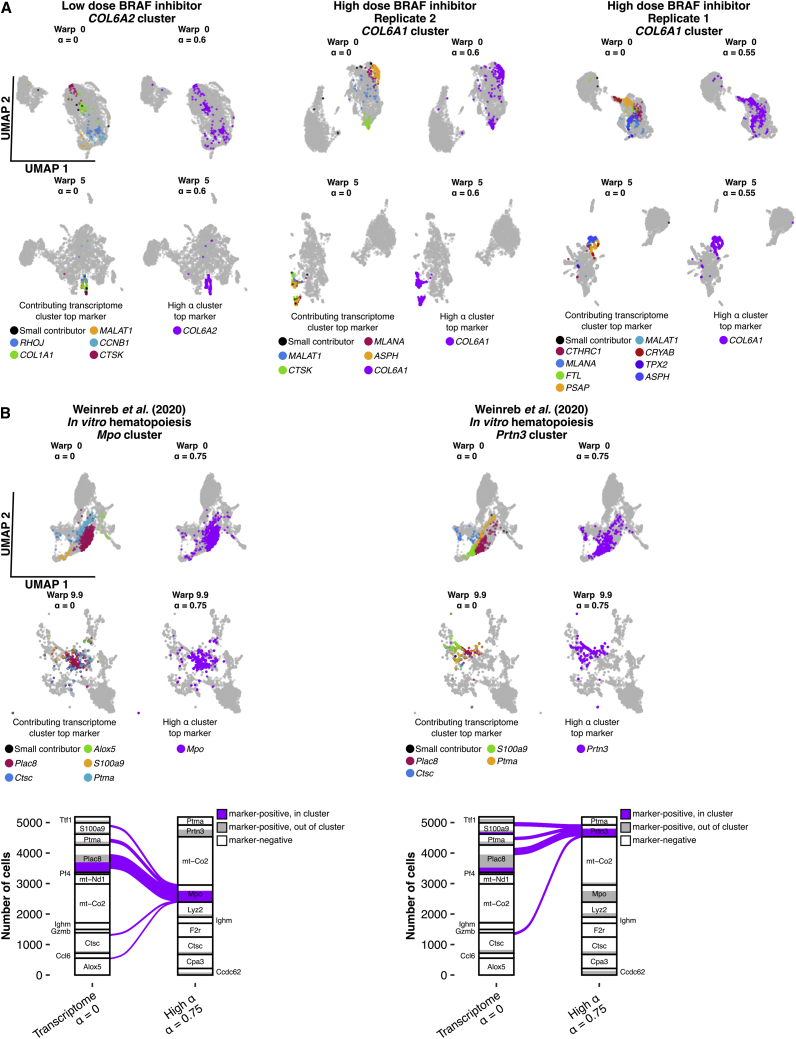
Combining hybrid clustering and Warp Factor highlights clusters with distinct markers in UMAP representations (A) UMAPs highlighting a single high α cluster with the indicated top marker gene (*COL6A1* and *COL6A2*) in different replicates and doses of WM989 cells treated with vemurafenib.^11^ Representations are stratified by α and Warp Factor. Gray points represent cells outside the high α cluster. Colors reflect the top marker gene of the high α cluster (purple) or the contributing transcriptome clusters. Contributing transcriptome (α = 0) clusters were grouped as “small contributors” if fewer than 10 cells were present in the high α cluster of interest. (B) UMAPS as in (A) for the day 2 *in vitro* hematopoiesis data^12^ highlighting high α clusters marked by the granulocyte-enriched markers *Mpo* and *Prtn3* (top) and Sankey diagram depicting marker positivity at both clustering levels, nodes are annotated with top cluster markers. “Small contributors” indicates all contributing transcriptome clusters that contributed fewer than 10 cells to the high α cluster.

We sought to demonstrate the potential biological utility of ClonoCluster and Warp Factor when used together, focusing on data from melanoma^11^ and hematopoiesis.^12^ Starting with melanoma, we first identified top marker genes at a high α (α from 0.55 to 0.6); these markers were distinct from those identified by purely transcriptomic clustering (Figure 5A). Among these were *COL6A2* in low-dose and *COL6A1* in high-dose vemurafenib treatment, which was consistent with our findings that collagen-associated gene sets were enriched in reorganization markers (Figure 3C) in the three replicates of low- and high-dose vemurafenib-treated WM989 cells.^11^ In the standard UMAP, cells that highly expressed these markers were dispersed, but upon engaging a Warp Factor of 5, these cells came together, demonstrating that Warp Factor can visually represent the results of incorporating clonal information.

We also performed a similar evaluation on the day 2 *in vitro* hematopoiesis data from Weinreb et al.^12^ The original authors labeled the majority of these cells as “undifferentiated” at day 2, as they did not meet the expression level for conventional markers used for supervised cutoffs to define cell types.^12^ Indeed, using conventional transcriptome clustering, many of the top cluster markers identified (*Alox5, Plac8, S100a9*, *Ptma,* and *Ctsc)* were not ones known to be associated with distinct cell types in this system. However, for high α (α = 0.75), two markers emerged as top cluster markers (*Mpo* and *Prtn3*) that are known neutrophil-specific genes (Figure 5B).^26^ Using a Warp Factor of 9.9 (likely needed due to the small number of cells per clone), we could pull these markers together to some degree.

The fact that ClonoCluster was able to recover more biologically meaningful markers suggests that there may be biological utility in its use. To further assess whether ClonoCluster generated more biologically meaningful groupings, we computed the entropy of distribution of the cells with known types based on cutoffs of marker genes. Lower entropy in this setting indicates that cells of the same type (biologically determined) are distributed across fewer clusters (computationally determined). We found that indeed, at low α, ClonoCluster reduced the entropy for several cell types (basophils, erythroid progenitors, lymphoid progenitors, mast cells, megakaryocytes, migratory dendritic cells, and plasmacytoid dendritic cells) as compared with clustering based on the transcriptome alone. As α was further increased, this decrease in entropy was often reduced or lost, suggesting that both clonal and transcriptomic information are needed to best reflect biologically meaningful clusters (Figure S7). The improvement in entropy for cluster distribution among cells of the same type alongside the identification of more specific markers is promising evidence that hybrid clonal-transcriptome clusters better reflect known biology.

## Discussion

Clonal barcoding has provided additional information that could be used for clustering cells based on both clonal origin and transcriptome. ClonoCluster provides a method for weighting clone and transcriptome information—using a tunable parameter α—to generate hybrid clusters. These clusters were distinct from purely transcriptomically defined clusters, with unique sets of marker genes. The reorganization was often accompanied by the alignment of the expression of extracellular matrix proteins, suggesting that category of protein may be important for discriminating clones from each other. Further, we developed Warp Factor, inspired by ClonoCluster, as a way to modify the popular UMAP visualization to incorporate clone information.

A major question is whether these hybrid clusters more accurately reflect biological differences than transcriptomically defined clusters. We propose ClonoCluster as a method to tune the degree to which clonal information is incorporated. There are suggestions that some degree of clone information does reveal biological information. First, the fact that the assignment of cells to clusters changes dramatically as α is changed suggests that there is at least different information captured by clone information. Moreover, the top markers for clusters changed markedly, again suggesting different biological characterizations. It is hard to know what markers are biologically “correct,” but we do point out that in the case of the *in vitro* hematopoiesis dataset, we found that increased α led to the detection of *Mpo* and *Prtn3* as markers, which are well-known neutrophil-specific genes^26^ that did not appear as a top cluster marker for pure transcriptomic clustering. Furthermore, we found that intermediate levels of α better recapitulated known biology by reducing the entropy of known cell types across computationally generated clusters. Moreover, in the case of cardiomyocyte-directed iPSCs from Jiang et al.,^18^ in which we know that cell fate is largely determined by extrinsic factors and thus less by clonal factors, we observed that marker fidelity decreased at the high α levels, providing a negative biological control for ClonoCluster. Our analysis demonstrates that in some systems, the addition of clonal information to clustering generates clusters that reduce the likely erroneous spreading of known cell types across groupings and yields markers that better correlate with known biology. Therefore, a thoughtful interrogation of the experimental system, considering the character of the markers identified in hybrid α clusters alongside the overall marker strength with increasing α, will help to determine if the addition of clonal information to clustering with ClonoCluster provides biologically meaningful insight. We expect that hybrid clustering will reveal important markers and groupings for many systems in developmental biology and other areas where clonality also seems to correlate with cell fate decisions that would be difficult to obtain using transcriptome-based clustering alone. Recent work by Wang et al.^27^ developing the Co-Spar algorithm robustly demonstrates the utility of incorporating clonal information to identify early fate biases in progenitor cells. The Co-Spar approach identifies clonal fates over multiple time points to approximate the transition manifold and assign likely progenitor states to even singly observed clones. This approach is powerful for identifying early states that commit to known fates by relying on data from multiple time points. In settings without such time series data, ClonoCluster provides a tunable time-independent approach to incorporating clonal and transcriptomic information into fate identification. Further development of tools will be needed as the number of experiments incorporating lineage tracing in different contexts continues to grow.

A potentially important consideration is whether the system in question is at an endpoint, or is still in the process of transitioning to its final state. It is in principle possible that the appropriate relative weighting between transcriptome and clone information may differ in these two contexts. Most likely, we think scenarios in which cell fate is determined by intrinsic states will benefit the most from ClonoCluster. That property may hold for both transitional or endpoint cell states, and hence will likely need to be evaluated on a case by case basis. Indeed, in our application of ClonoCluster to two transitional cell systems, hematopoiesis and directed cardio-myocyte differentiation, the former was intrinsically determined and ClonoCluster was informative, whereas the latter showed extrinsic determination and ClonoCluster was less informative. Consequently, we think that “twin” analysis (barcoded twin cells subjected to different local environments^11^) are critical controls that should be performed to determine the relative contributions of intrinsic and extrinsic factors to help judge whether ClonoCluster will be a useful algorithm to apply.

What are the primary transcriptomic correlates with the rearrangements driven by incorporating clone information? We found these correlates were primarily associated with translation, ribosomal activity, and components of the extracellular matrix. These associations were found across diverse, independent datasets, suggesting that it is a common source of variation across the transcriptomes of clones. There is a known association between ribosomal gene expression and overall gene expression level that may be the source of its influence on cell clustering.^6^ Extracellular matrix may indicate some memory of initial microenvironmental differences that differ between clones. This memory would persist independent of subsequent microenvironmental differences, however, because “twin” experiments have shown that the same clone exposed to two different microenvironments post-drug have virtually identical transcriptomes.^11^ It is thus also possible that extracellular matrix proteins inherently reflect stable cell type or state identifiers.^28^ It is also perhaps surprising that clonal information is carried by these sets of factors that are not transcription factors, which are more canonically thought of as the critical regulators of cell type. That difference suggests that extracellular matrix proteins may be more important for these subtle aspects of cell type determination than generally appreciated.

In general, the problem we describe here is in many ways analogous to whether species should be organized by genetic phylogeny or by phenotypic characteristics.^29^ In some ways, categorizing species by phenotype would be analogous to clustering cells by their phenotype (i.e., transcriptome), whereas categorizing species by genetic phylogeny would be akin to clustering cells by lineage. Early zoology, predating genetic information, categorized species by observable phenotypes, but the addition of genetic information quickly grew to be an important form of categorization; by analogy, using clone information in cell clustering may similarly alter our classifications of cell types.

### Limitations of the study

Ultimately, further testing of the homogeneity in the functional properties of cells within each cluster will be required to truly determine what clustering methods most closely match biological distinctions. Our studies did not reveal a reliable method to identify which experimental systems have intrinsic determinants of cell fate and therefore are most likely to yield more biologically meaningful clusters with the addition of clonal information. For now, we recommend an empiric approach to determine if the addition of clonal information is useful by first identifying the resolution value that returns the desired number of clusters from standard transcriptome-only clustering, and then plotting the number of clusters returned with increasing α. As α approaches 1, the number of hybrid clusters approaches the number of unique clone barcodes in the data. This visualization allows the user to determine the maximum α value that the experimental system will tolerate to generate a comparable number of clusters to transcriptome-only clustering while incorporating lineage information. Analysis of marker fidelity or comparison to ground truth data can be used to confirm that the α value produces hybrid clusters that are biologically meaningful. Example code to determine the optimal α value and an example protocol is provided in the *ClonoCluster* GitHub repository (https://github.com/leeprichman/ClonoCluster/blob/main/Tutorial.Rmd; https://doi.org/10.5281/zenodo.3369197.)

Furthermore, technology for reading out full-lineage data (as opposed to just clone data) has now been developed, often using CRISPR-Cas9 genome editing to make mutations in the genome that can be read out by sequencing^30^^,^^31^^,^^32^^,^^33^ or imaging to generate the full cellular family tree.^33^^,^^34^^,^^35^^,^^36^ Our analysis did not address these types of data, although it is relatively straightforward to adapt ClonoCluster to these situations, in which one can weight the distance between cells by the relative phylogenetic distance. We expect that full-lineage tracing with time series information rather than the limited-resolution single time point data we explored will increase in prevalence, and ClonoCluster and like methods will need to adapt to this higher resolution setting.

## STAR★Methods

### Key resources table

**Table:** 

REAGENT or RESOURCE	SOURCE	IDENTIFIER
**Deposited data**		

WM989 melanoma cells treated with vemurafenib, WM983b melanoma cells treated with vemurafenib, MDA breast cancer cells treated with Paclitaxel	Goyal et al., 2021^11^	https://www.biorxiv.org/content/10.1101/2021.12.08.471833v1
Murine hematopoietic stem cells *in vitro* differentiation	Weinreb et al., 2020^12^	https://www.science.org/doi/10.1126/science.aaw3381
Human induced pluripotent stem cell line directed toward cardiomyocyte fate	Jiang et al., 2022^18^	https://genomebiology.biomedcentral.com/articles/10.1186/s13059-022-02654-6

**Software and algorithms**		

*ClonoCluster* (https://doi.org/10.5281/zenodo.3369197)	This paper	https://github.com/leeprichman/ClonoCluster (https://doi.org/10.5281/zenodo.3369197)
*Seurat*	Satija et al., 2015^19^	https://cloud.r-project.org/package=Seurat
*WebGestaltR*	Liao et al., 2019^24^	https://cran.r-project.org/web/packages/WebGestaltR/index.html
*data.table, entropy, magrittr, ggplot2, devtools*	Comprehensive R Archive Network	https://cran.r-project.org/

**Other**		

Analysis scripts, count matrices, barcode matrices, and output (https://doi.org/10.5281/zenodo.7105982)	This paper	https://github.com/arjunrajlaboratory/ClonoCluster_paper (https://doi.org/10.5281/zenodo.7105982)

### Resource availability

#### Lead contact

Further information and requests for resources and reagents should be directed to and will be fulfilled by the lead contact, Dr. Arjun Raj (arjunraj@seas.upenn.edu).

#### Materials availability

This study did not generate new unique reagents.

### Method details

#### Transcriptome and clone barcode integration with ClonoCluster

To integrate the transcriptome and clone barcode information, the shared nearest neighbors network graph is produced, using the single cell RNA sequencing analysis R package *Seurat*.^19^ Briefly, gene counts are converted into 100 principal components using the *irlba* package.^38^ *k* nearest neighbors in PCA space are identified and a network graph is constructed where cells are nodes and edges between any two cells are given weight equivalent to the jaccard index between shared nearest neighbors (defined below as *J*). For all analyses, *k* was set to 20 and the graph was pruned of any edges with weight less than 1/15, consistent with *Seurat* defaults.

A size-normalized clone barcode network graph is also constructed where each cell is represented as a node, with edges drawn between all cells with weights of 0 for cells that are not in the same clone cluster (i.e. do not share a barcode) or 1/*n* for cells that are in the same clone cluster (i.e. do share a barcode), where *n* is equal to the total number of cells assigned to that barcode (size of the clone cluster.) We found that size normalization is necessary to prevent the highly interconnected larger clone clusters from exerting a dominant effect on graph modularity and final clustering compared to smaller clone clusters.

The transcriptome and clone graph edge weights are integrated using the following equation:

$$W=\alpha^{\beta }\left(lin-J\right)+J$$

Where *W* is the final output edge weight between two cells, α is a chosen parameter ranging from 0 to 1, β is a chosen parameter from 0 to 1, *lin* is the value of the edge in the clone barcode graph {0,1/*n*} and *J* represents the edge weight in the transcriptome graph: the jaccard index of shared nearest neighbors in transcriptome space. At α = 0, the edge weight between any two cells is therefore equivalent to the transcriptome graph (*J*), and at α = 1 the edge weight is equivalent entirely to the clone barcode graph (*lin*). β modifies the effect of step sizes in α, lower values of β increase the effect of α on final edge weight *W* at low α. For all datasets and analyses, β was set to 0.1. The combined graph with modified edge weights is then passed to the Louvain community detection algorithm^22^ as implemented in *Seurat* to return cluster assignments.

#### Cluster assignments

Resolution for the community detection algorithm was fixed for each sample, ranging between 0.6 and 1 across all nine replicates, with an initial target of 8–20 clusters at α = 0. As α approaches 1, the number of clusters returned at fixed resolution approaches *n*, the number of unique clone barcodes present. Therefore, the “high α” level was identified by approximation of the α value at the inflection point where the number of clusters rapidly increases and begins to reflect single clone clusters. The “low α” level was defined as half the “high α” value rounded to the nearest tenth. This gave the maximum number of clusters as 32 at the high α level in the Jiang et al.^18^ dataset.

#### Clone barcode to cluster correlation analysis and visualization

Sankey diagrams were generated using the *ggalluvial* R package. For Sankey diagrams inclusive of α = 1, the top 15 largest clone clusters per sample are chosen for ease of visualization and limitation of discrete colors used. Unless otherwise specified, the complete dataset is shown for Sankey diagrams. Cohen’s κ was computed at each α level per clone barcode for the hybrid cluster with the largest proportion of the barcoded cells within it.

#### Cluster marker identification

Cluster markers were identified by receiver operating characteristic (ROC) using the *ROCR* package.^39^ All genes present in the count matrix were tested for classification of a cell within a cluster versus all other clusters combined. When cells are noted as “marker positive”, the threshold for positivity was identified from the point on the ROC curve with minimum Euclidean distance from 100% TPR and 0% FPR.

#### Reorganization marker and overrepresentation analysis

For each cluster of interest at a given α value, cells from each contributing cluster at the transcriptome level (α = 0) were compared to all other cells from the contributing transcriptome cluster. ROC analysis was performed to determine the strength of genes in the count matrix to classify cells from the contributing transcriptome cluster that reorganize to the α cluster of interest as long as the two comparison groups contained at least 10 cells. The AUC from this analysis was termed the reorganization AUC or reorg-AUC. Reorganization marker genes were defined as genes with reorg-AUC >80 (corresponding to a “large” effect size.)^40^ This stringent threshold was chosen to eliminate potentially spurious markers and more accurately reflect the true biology, maximizing the usefulness of downstream gene set level analyses. Each set of reorganization markers for the paired hybrid α cluster and contributing transcriptome (α = 0) cluster was used for overrepresentation analysis. Overrepresentation analysis is a gene set analysis approach that identifies whether a given list of unranked differentially expressed genes is enriched for members of known gene sets representing biological processes or pathways, such as those from the Gene Ontology Consortium,^41^ compared to selection at random. When a transcriptome cluster contributed in its entirety to an α cluster (i.e. the transcriptome cluster was a subset of the α cluster), the AUC for overall cluster markers for the contributing transcriptome cluster was considered the reorg-AUC. Thresholds for marker positivity for visualization were determined from the point on the ROC curve with minimum Euclidean distance from 100% TPR and 0% FPR.

Overrepresentation analysis was performed with the R package *WebGestaltR.**^24^* The search space included the Gene Ontology Cellular Components and Molecular Function datasets.^41^ Significantly overrepresented gene sets were defined as those with FDR <0.05. For visualization, heatmaps were created of the maximum log base 2 of the enrichment ratio for any cluster reorganization in the sample.

#### Modified principal components and UMAP visualization

Uni-form manifold approximation and projection (UMAP) is a dimensionality reduction technique frequently used to visualize gross trends in high dimensional single cell RNA sequencing data.^25^^,^^42^ Clustering assignments and associated markers computed from principal component space are usually overlaid as well.^6^ To incorporate clone barcode information into the two dimensional UMAP representation of data, we modified the principal components using a tunable parameter *s* according to the following equation:

$$M_{s}=M-\frac{s}{10}*\left(M-M_{b}\right)$$

Where *M*_*s*_ is the modified PCA matrix passed to the UMAP algorithm, *M* is the PCA matrix, *s*, Warp Factor, is a parameter from 0 to 10, and *M*_*b*_ is a matrix composed of the average value of the PCA matrix per clone. At *s* = 0, the input to the UMAP will be the PCA matrix, *M*. At *s* = 10, the input to the UMAP will be a matrix with the value of each principal component substituted for the corresponding mean value per clone barcode, *M*_*b*_, thus the only variation reflected in the UMAP will be between clones (Figure S5B). The parameters for UMAP as implemented in the *uwot* package were identical to the default settings of *Seurat*.^19^^,^^42^

### Quantification and statistical analysis

Data manipulation and statistical analysis was performed in the R statistical computing language with the *data.table, stats*, *ROCR, WebGestaltR*, and *magrittr* packages. Entropy was calculated with the *entropy* package using the Laplace method. Transcriptome nearest neighbor graph construction and community detection was performed with the *Seurat* package. Data visualization used the R packages *ggplot2*, *pheatmap*, *ggalluvial*, and *VennDiagram*. Global hypothesis testing was performed using the non-parametric Kruskal-Wallis test with Bonferroni correction for multiple comparisons. Local testing was performed with pairwise Wilcox tests with Bonferroni correction.

## Data Availability

• Single cell RNA sequencing and barcode data for six datasets were compiled from multiple sources. “Low dose BRAF inhibitor” and “high dose BRAF inhibitor” indicate replicates of a monoclonal human melanoma cell line, WM989 A6-G3,^14^ transfected with a barcode library and treated with 250 nM and 1μM vemurafenib respectively. “WM983B BRAF inhibitor” samples indicates replicates of a vemurafenib-resistant monoclonal human melanoma cell line, WM983B E6-C6^14^ transfected with a barcode library and treated with 250 nM vemurafenib.^11^ “Paclitaxel-treated breast cancer cells” indicates replicates of a monoclonal line, MDA-MB-231D4, derived from human breast cancer cell line transfected with a barcode library and treated with 1 nM paclitaxel, as previously described.^11^^,^^37^ “Cardio-directed iPSC” indicates a barcode-transfected induced pluripotent stem cell line treated to drive cells toward a cardiomyocyte fate as previously described.^18^ Count matrices, barcoding methods, and barcode assignments for these samples were generated as described.^11^^,^^18^ “*In vitro* hematopoiesis” samples indicate barcoded murine hematopoietic stem cells differentiating *in vitro*, collected and sequenced at the day 2 time point when most cells were labeled “undifferentiated” using known cell markers. Count matrices and barcode assignments were deposited by Weinreb et al.^12^ and retrieved from the NCBI Gene Expression Omnibus at https://www.ncbi.nlm.nih.gov/geo/query/acc.cgi?acc=GSE140802. Count matrices and barcodes for all datasets used in this study are available in the analysis repository on GitHub at https://github.com/arjunrajlaboratory/ClonoCluster_paper (https://doi.org/10.5281/zenodo.7105982). • Raw data and scripts for all analyses are available at https://github.com/arjunrajlaboratory/ClonoCluster_paper (https://doi.org/10.5281/zenodo.7105982). ClonoCluster is open source and available under the GPL3 license at https://github.com/leeprichman/ClonoCluster (https://doi.org/10.5281/zenodo.3369197), including worked examples, example data, unit tests, and a Docker image. • Any additional information required to reanalyze the data reported in this paper is available from the lead contact upon request.

## Supplemental information

Document S1. Figures S1–S7

Document S2. Article plus supplemental information
