# Supplementary material for: ClonoCluster: A method for using clonal origin to inform transcriptome clustering
Source: Cell Genom. 2023 Jan 12;3(2):100247. doi: 10.1016/j.xgen.2022.100247 (PMC9932990; doi:10.1016/j.xgen.2022.100247)
Supplement: Document S1. Figures S1–S7 [file mmc1.pdf]

Cell Genomics, Volume *3*

Supplemental information

ClonoCluster: A method for using clonal origin to inform transcriptome clustering

Lee P. Richman, Yogesh Goyal, Connie L. Jiang, and Arjun Raj

Supplemental Figure 1

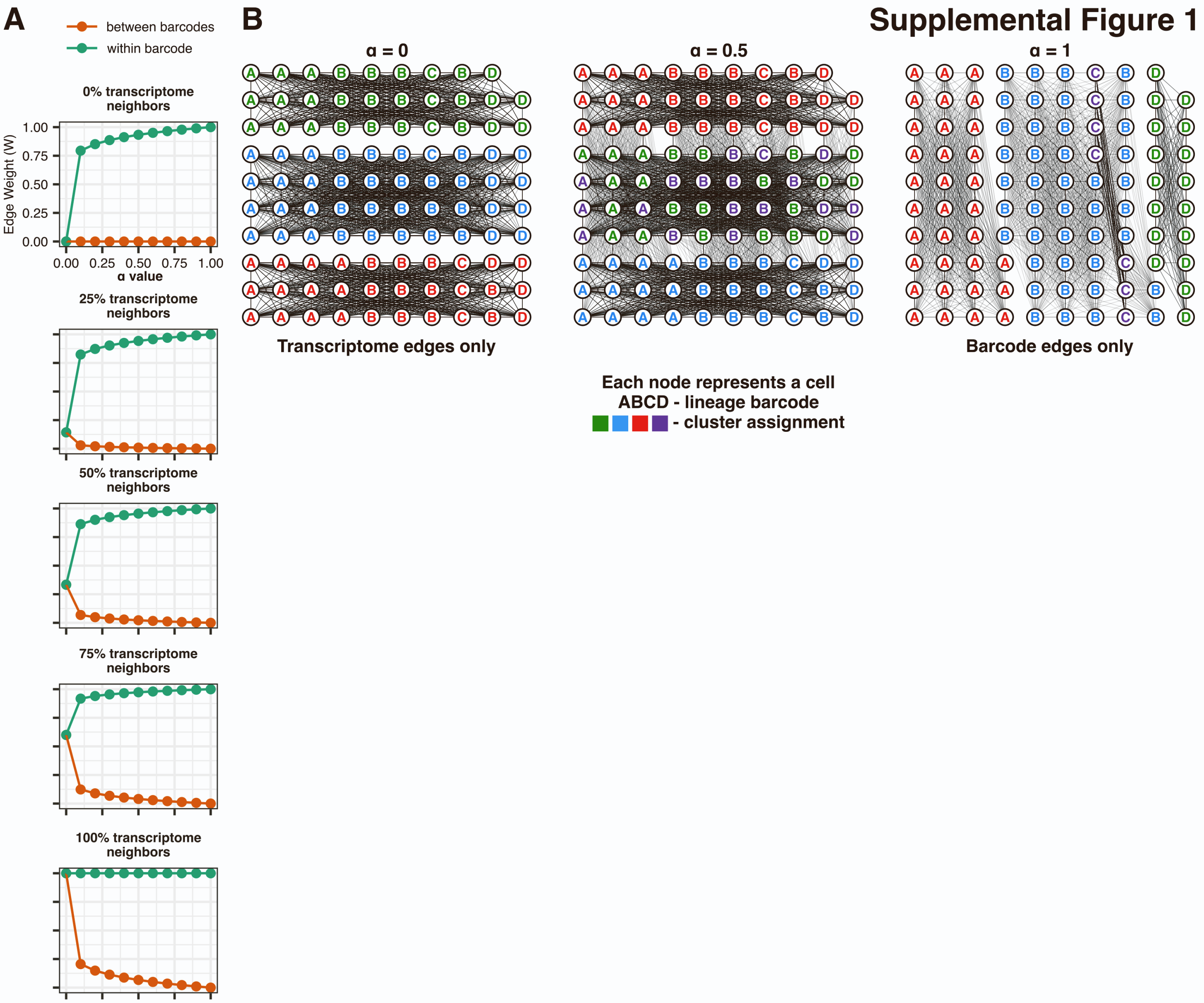

Figure S1, Related to Figure 1: α modifies network graph edge weights to create Louvain clusters that increasingly reflect clone barcode assignments.

A. Output edge weight of the ClonoCluster model for graph edge weights between two cells for different proportions of shared nearest neighbors in transcriptome space with fixed ß = 0.1.

B. Network graphs with modified edge weights and cluster assignments at three α values for simulated data.

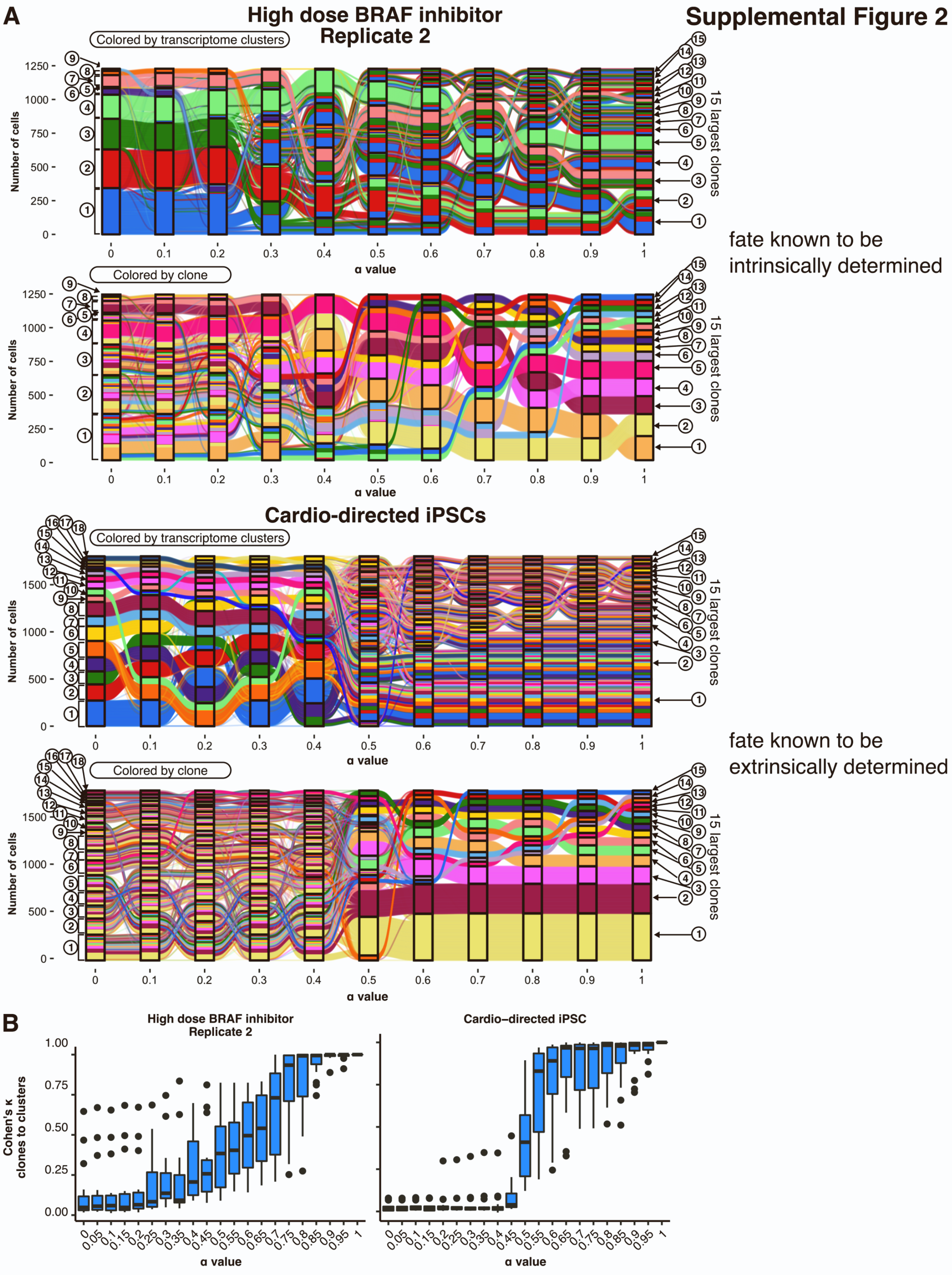

Figure S2, Related to Figure 1: Intrinsic vs extrinsic determinants of cell fate stratify the effect of α on clone-cluster congruence.

A. Sankey diagrams for the 15 largest clone clusters in two samples across α values, a clonal melanoma cell line treated with high dose (1μM) vemurafenib [11] with known intrinsic determinants of cell fate and directed differentiation of induced pluripotent stem cells (iPSCs) towards a cardiomyocyte fate [18] with previously described dominance of extrinsic determinants of cell fate.

B. Cohen's κ for interrater reliability for classification of a cell by clone barcode and cluster assignment for the top 15 largest clone clusters across α values for the samples in **(A)**.

Supplemental Figure 3

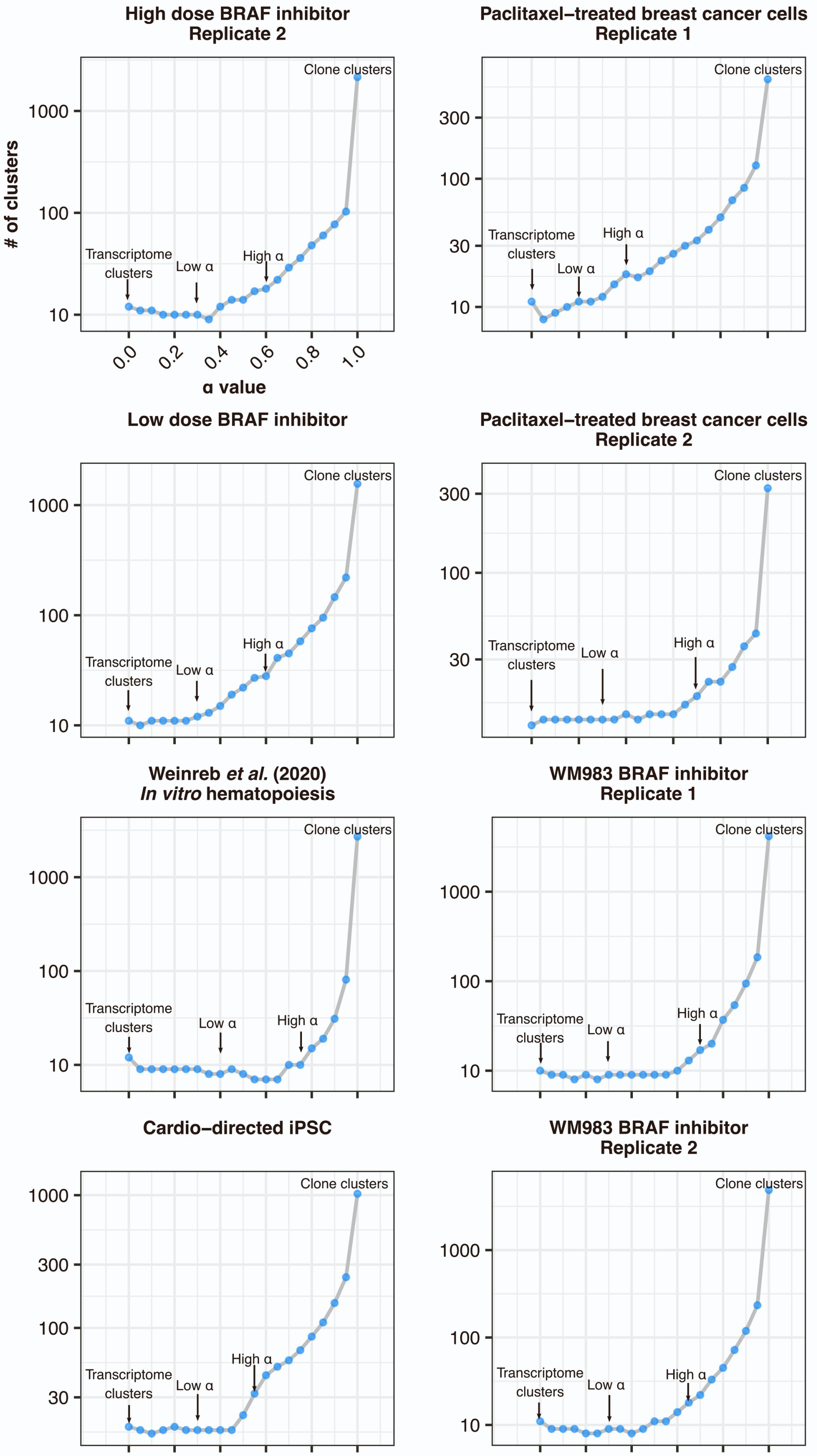

Figure S3, Related to Figure 1: The number of clusters returned by community detection at fixed resolution approaches the number of unique clone barcodes in the data as α approaches 1.

Plots showing the number of clusters returned by community detection at constant resolution with increasing α in each dataset. At α = 1, the network graph is composed of like-barcoded cells (a clone cluster) connected only to each other and community detection returns the number of unique clone clusters in the data as the number of clusters.

Supplemental Figure 4

Low dose BRAF inhibitor
top cluster markers

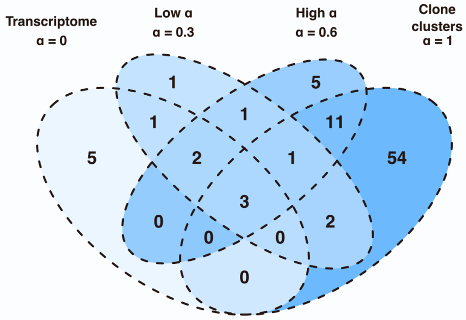

High dose BRAF inhibitor
Replicate 1
top cluster markers

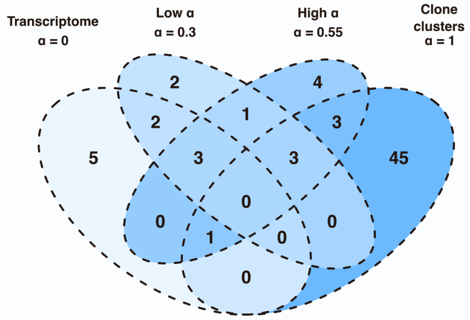

High dose BRAF inhibitor
Replicate 2
top cluster markers

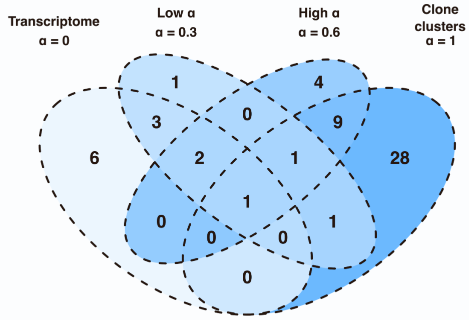

Figure S4, Related to Figure 2:
Significant marker turnover occurs between transcriptome, low α, high α, and clonal clusters.
Venn diagrams showing the number and overlap of markers with AUC > 0.70 for any transcriptome, low α, high α or clone cluster.

Weinreb *et al.* (2020)
***In vitro* hematopoiesis**
top cluster markers

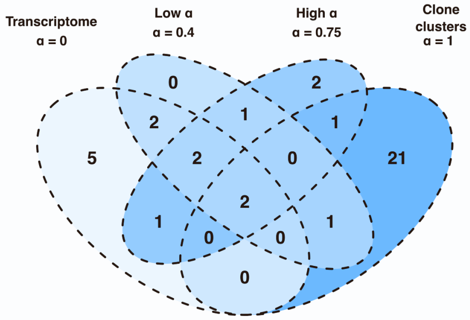

Cardio–directed iPSC
top cluster markers

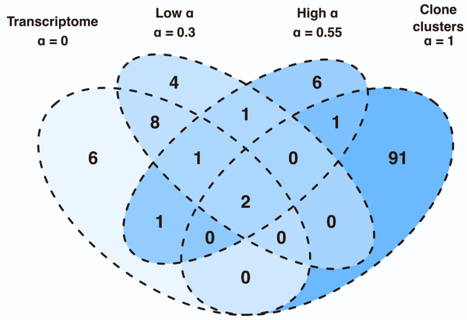

WM983B BRAF inhibitor
Replicate 1
top cluster markers

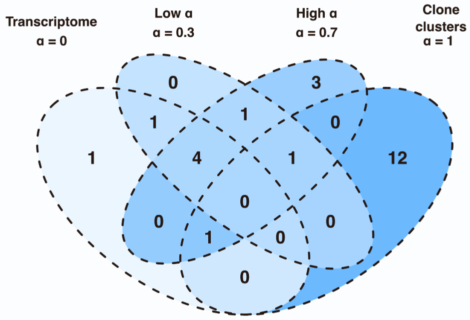

WM983B BRAF inhibitor
Replicate 2
top cluster markers

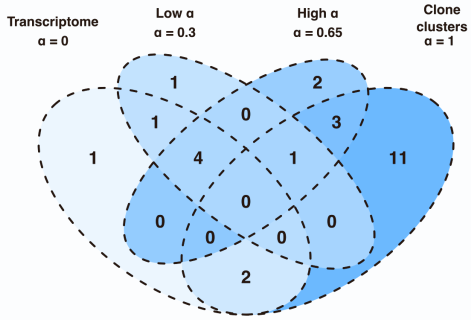

Paclitaxel–treated breast cancer cells
Replicate 1
top cluster markers

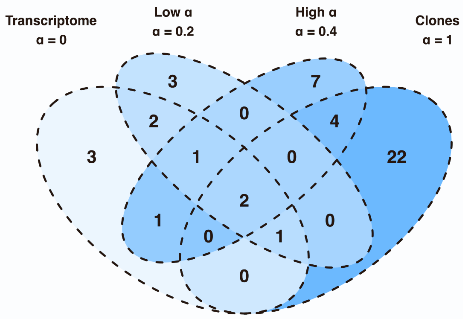

Paclitaxel–treated breast cancer cells
Replicate 2
top cluster markers

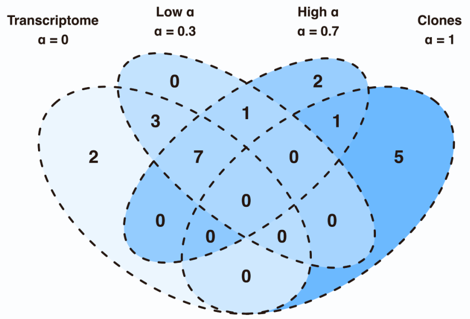

Supplemental Figure 5

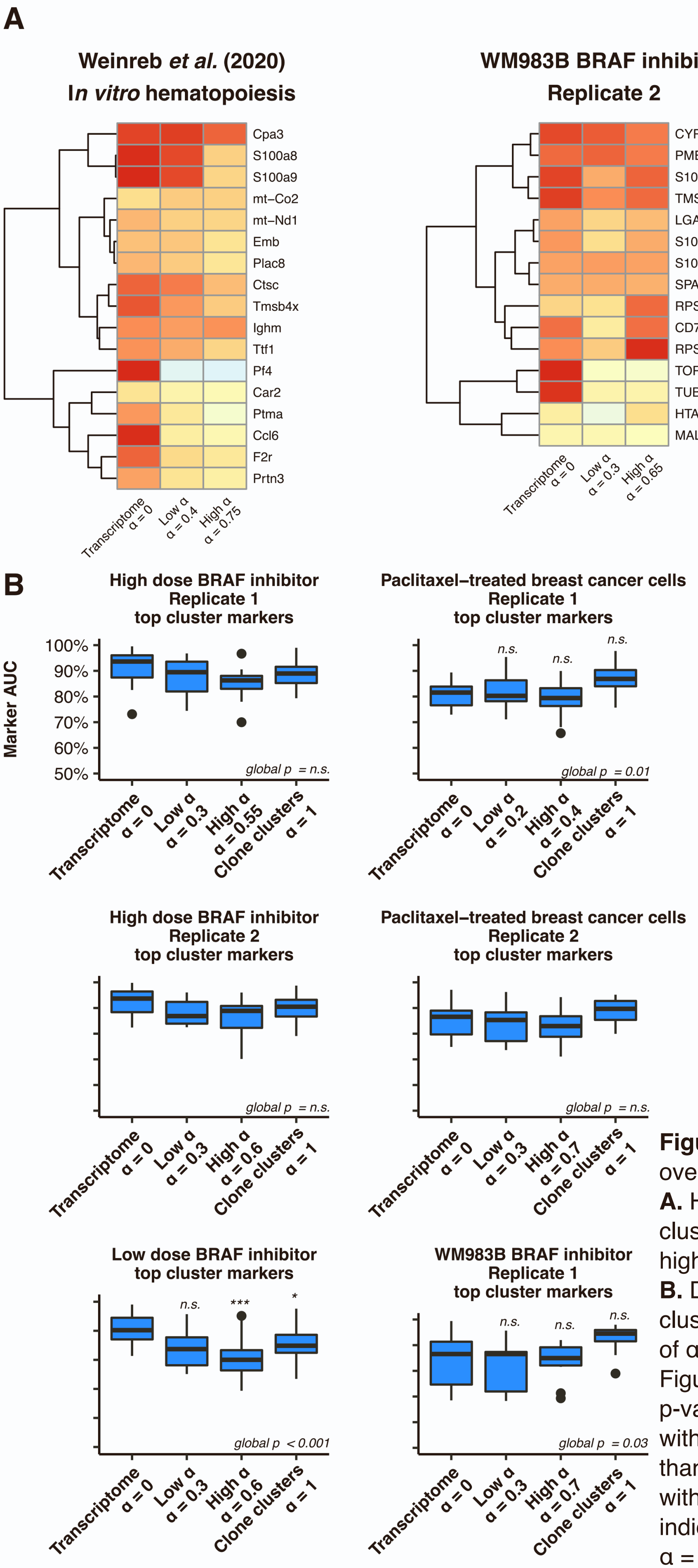

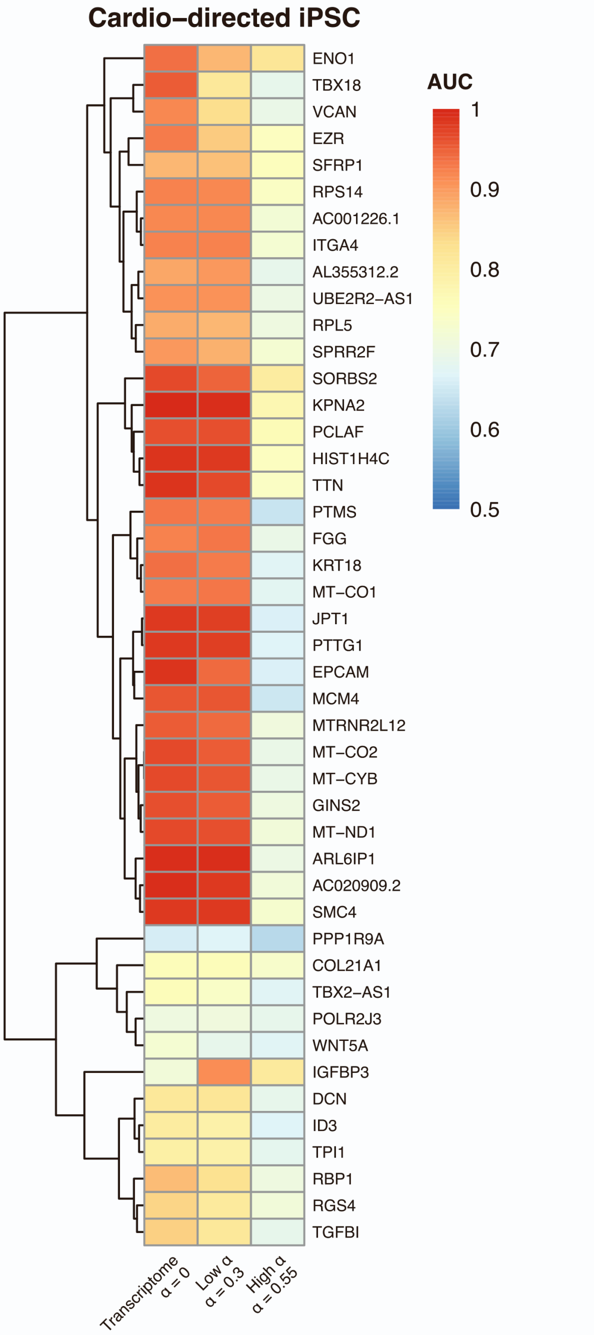

Figure S5, Related to Figure 2: Top cluster marker overall fidelity is preserved across hybrid clusters
A. Heatmaps of AUC values for the union of all top cluster markers at the transcriptome, low α, and high α level.
B. Distributions of AUC values for the top marker per cluster at zero, low, high, and maximum (α = 1) values of α for the remaining samples not depicted in Figure 2D (see Methods). "Global p" indicates the p-value for the non-parametric Kruskal-Wallis test with Bonferroni correction. When "global p" was less than <0.05, pairwise Wilcox tests were performed with Bonferroni correction. Annotations above boxes indicate the corrected Wilcox test p-value compared to α = 0 (n.s. - not significant, * - p < 0.05, *** - p < 0.001).

A

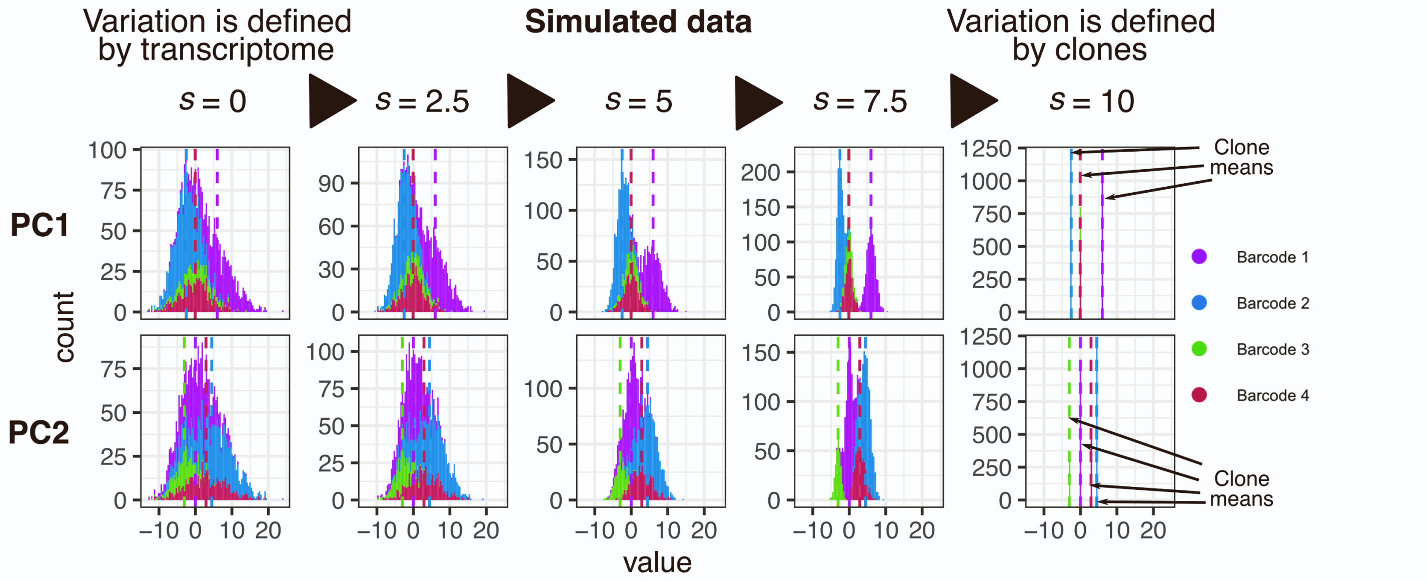

$$M_s = M - \frac{s}{10} * (M - M_b)$$

M_s – Warped PC matrix passed to UMAP
M – Matrix of PCs
s – scalar from 0 to 10
M_b – Matrix of mean PC values by barcode

B

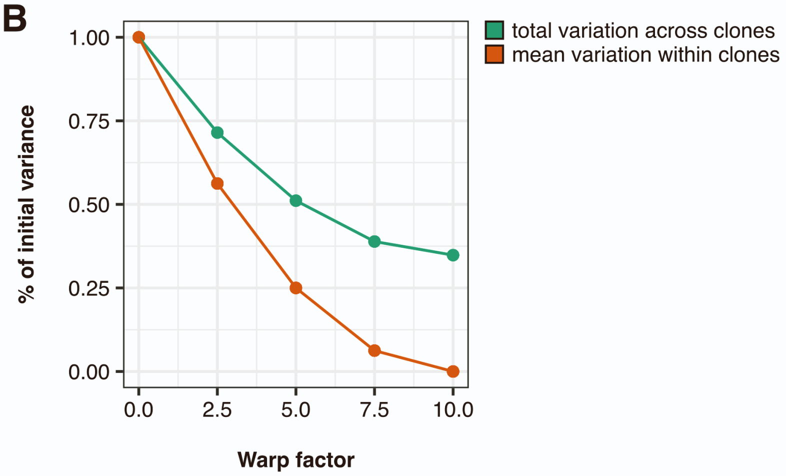

Figure S6, Related to Figure 4: Increasing Warp Factor, s, reduces the variation of principal component values around clone barcode means.

A. Histograms colored by clone barcodes demonstrating the distribution of PC values for two principal components for simulated data for 3,000 cells with four clone barcode assignments. Vertical dashed lines represent the mean values for each clone barcode at s = 0, equivalent to the unmodified PC means. At s = 10, modified PC values are equivalent to the assigned clone barcode means. Modified PC values are then passed to the UMAP implementation for visualization as in Figure 4.

B. Mean variation of all PCs in simulated data within and between clone barcodes stratified by values of s.

Supplemental Figure 7

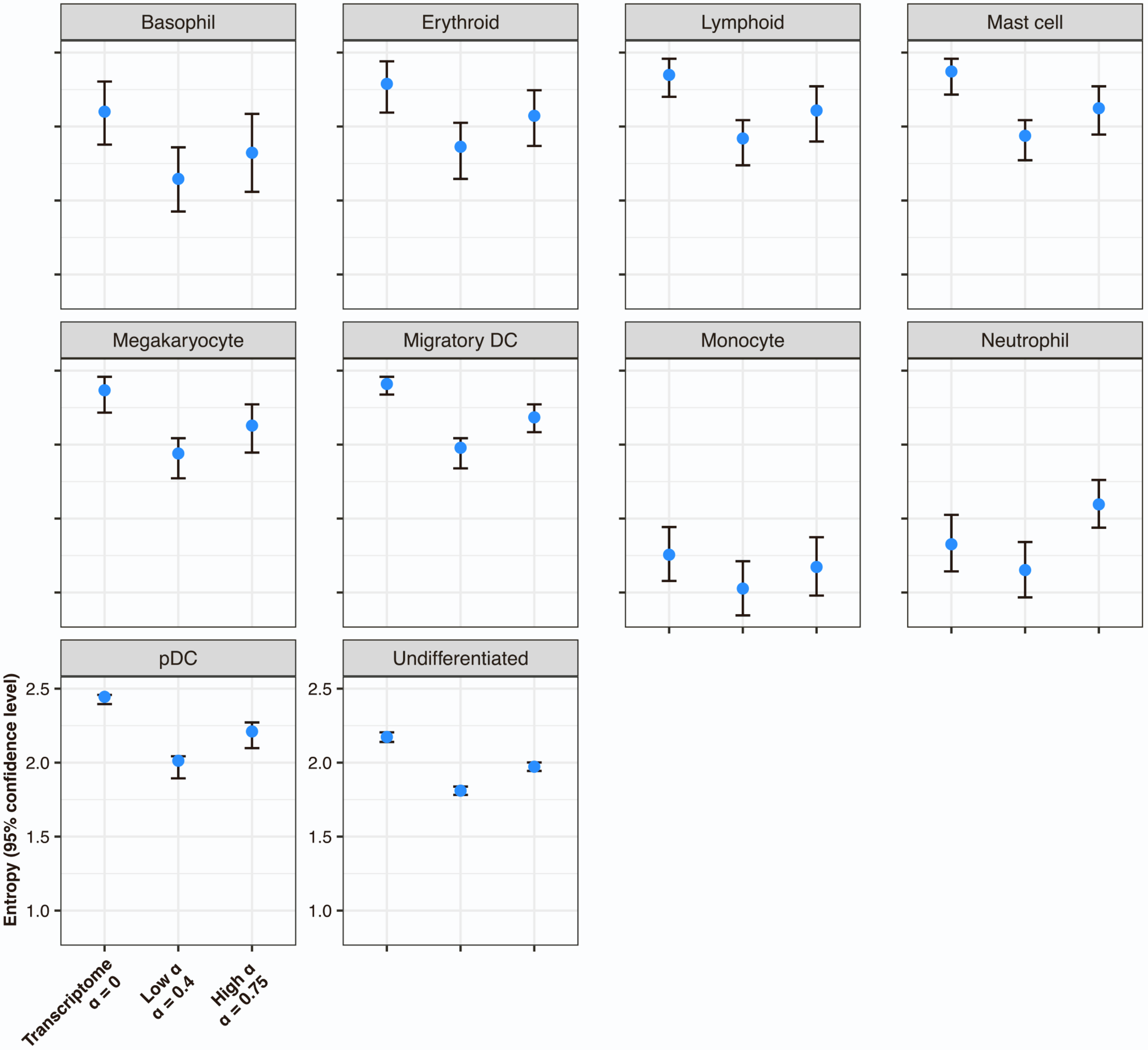

Figure S7, Related to Figure 5: Low α clusters reduce entropy of clustering for multiple hematopoietic cell types. Laplace entropy was calculated for the distribution of known cell types in hematopoiesis data[12] across transcriptome-only clusters, low α, and high α. Entropy is shown in bits, error bars represent 95% confidence intervals using the Monte Carlo method with 1,000 samplings of 33% of the cells.
